# Clinical Outcome After Pencil Beam Scanning Proton Therapy of Patients With Non-Metastatic Malignant and Benign Peripheral Nerve Sheath Tumors

**DOI:** 10.3389/fonc.2022.881665

**Published:** 2022-06-27

**Authors:** Nicolas Bachmann, Dominic Leiser, Alessia Pica, Barbara Bachtiary, Damien C. Weber

**Affiliations:** ^1^ Center for Proton Therapy, Paul Scherrer Institute, ETH Domain, Villigen, Switzerland; ^2^ Department of Radiation Oncology, Inselspital, Bern University Hospital, Bern, Switzerland; ^3^ Department of Radiation Oncology, University Hospital of Zürich, Zürich, Switzerland

**Keywords:** malignant peripheral nerve sheath tumors, proton therapy, pencil beam scanning, adolescents and young adults, neurofibromatosis type 1, benign peripheral nerve sheath tumors

## Abstract

**Objective:**

Peripheral nerve sheath tumors (PNSTs) commonly arise from peripheral nerve roots and grow locally invasive. Malignant PNSTs (mPNSTs) represent aggressive sarcomas of neural origin that can originate from PNSTs. Radiation therapy is commonly used as part of the required multimodal treatment. However, both entities tend to occur early in life and are associated with the genetic disorder neurofibromatosis type 1 (NF-1), which is known to cause increased radiosensitivity. Pencil beam scanning proton therapy (PBSPT) allows for a minimization of the dose delivered to organs at risk and the integral dose and, thus, potentially also a reduction of radiation-induced adverse events. We report the clinical outcome and toxicity rates of patients with (m)PNSTs treated with PBSPT.

**Methods:**

We retrospectively reviewed 36 patients who received PBSPT (median dose, 64 Gy_RBE_) with curative intent for (m)PNSTs between 1999 and 2020 at our institute. Twenty-eight (78%) and 8 (22%) patients were treated at diagnosis and for tumor recurrence/progression, respectively. The median age was 32 years (range, 3–75), and 25 (69%) patients were male. mPNST and PNST were diagnosed in 31 (86%) and 5 (14%) patients, respectively. Underlying NF-1 disease was found in 8 (22%) patients. Acute and late toxicities were recorded according to Common Terminology Criteria for Adverse Events, version 4.1 (CTCAE v4.1). Overall survival (OS), local control (LC), and distant control (DC) were estimated using the Kaplan–Meier method.

**Results:**

With a median follow-up time of 31 months (range, 4–194), 13 (36%) patients died from a progressive disease, 8 (22%) experienced local failure, and 14 (39%) experienced distant failure after PBSPT. Estimated 2-year OS, LC, and DC were 75.5%, 73.5%, and 61.2%, respectively. Acute grade 3 toxicity (dermatitis, mucositis, and pain) was observed in 5 (14%) patients. Late grade 3 cataract and osteonecrosis were both observed in 1 (3%) patient at 34 and 194 months after PBSPT, respectively. There was no late grade >3 toxicity or radiation-induced secondary cancer.

**Conclusion:**

To our knowledge, this is the first study to analyze the outcome of (m)PNSTs treated with proton therapy using a PBS delivery paradigm. In our cohort, consisting mainly of patients with mPNSTs, we report reasonable oncological outcomes and low toxicity rates after PBSPT.

## Introduction

Peripheral nerve sheath tumors (PNSTs) and malignant PNSTs (mPNSTs) are neoplasms arising from peripheral nerves and are frequently also described as neurofibroma or schwannoma (PNST), neurofibrosarcoma, neurogenic sarcomas, or malignant schwannomas (mPNST). Both entities are often associated with the genetic disorder neurofibromatosis type 1 (NF-1) (1). PNSTs usually arise from Schwann cells (2) and are typically of benign character but can cause progressive and uncontrolled pain, neurologic deficits, compression/destruction of vital structures, and severe disfigurement (1). mPNST is a rare and highly aggressive soft tissue sarcoma of neural origin with an incidence of 1.46 per 1,000,000 person-years (3). mPNSTs account for approximately 10% of all soft tissue sarcomas and can originate from precursor PNSTs, particularly from plexiform neurofibromas (2, 4–10). An NF-1 disorder is found in approximately 50% of mPNST cases, 10% of all mPNSTs are radiation-induced, and the remaining 40% occur sporadically (6). Tumors of the peripheral nerves tend to present earlier in life (1, 4, 11, 12) than most other sarcomas, and mPNSTs represent one of the most frequent non-rhabdomyosarcomatous soft tissue sarcomas in the pediatric population (4, 13). A multimodal treatment approach including surgery and radiotherapy, with or without chemotherapy, is often administered due to the local aggressiveness and high potential to metastasize (14). Despite aggressive treatment regimens, the outcome for most patients remains poor, with median 5-year survival rates ranging from 15% to 50% (1). Studies have shown an increased radiosensitivity in NF-1 patients, which can lead to increased side effects from irradiation and a higher risk of secondary tumor induction (15, 16). With proton therapy (PT), the dose to organs at risk (OARs) can be reduced, and hence a lower integral dose is achievable. Considering that PNSTs/mPNSTs are often in a critical location (i.e., head or spine) and the young age of patients, PT seems an appropriate treatment strategy for this patient group.

The aim of this study is to report the oncological outcome and toxicity after pencil beam scanning PT (PBSPT) for PNSTs and mPNSTs and to assess the major prognostic factors for these challenging tumors.

## Materials and Methods

### Patients

Medical records of all non-metastatic patients who were treated with PBSPT for a PNST or an mPNST with curative attempt between 1999 and 2020 at our institution were retrospectively reviewed. Peripheral schwannoma, benign neurilemmoma, neurofibroma, and benign PNST were considered PNST. Malignant schwannoma, malignant neurilemmoma, triton tumor, malignant perineurioma, neurogenic sarcoma, neurofibrosarcoma, and mPNST were categorized as mPNST.

Patients with any age (pediatric patients <18 years, adolescents and young adults (AYA) 18–39 years, and adults >39 years) and any Karnofsky performance status (KPS for adults and AYA) or Lansky score (for pediatric patients) were included. Previous photon irradiations and combined treatments with protons and photons were allowed. Out of 164 patients screened in our institutional database, 1 patient with no follow-up data, 121 patients with inappropriate histology, and 6 patients with suspected PNSTs of cranial nerves diagnosed only radiologically were excluded. In total, 36 patients were included in the analysis. Approval from the competent ethics committee was obtained for this study (*Ethikkommission Nordwest- und Zentralschweiz 2021-00369*).

### Proton Therapy

Patients treated in 1999 and early 2000 received gantry-delivered PBSPT with a beam from the main 590-MeV cyclotron. From mid-2000 onward, PBSPT was delivered with an energy-degraded beam from a 250-MeV cyclotron. PBSPT was delivered as neoadjuvant, adjuvant, or definitive treatment for primary or recurrent tumors.

Treatment planning was performed either on the in-house planning system *PSIplan* or *Eclipse^®^* (Varian Medical Systems, Palo Alto, CA, USA). Multi-field optimization (MFO) and single-field optimization (SFO) techniques were used. For patients who underwent macroscopic complete tumor resection, the initial gross tumor was contoured in pre-surgery images (PET-CT, CT, and MRI). The pre-surgery images were fused with the PT planning-CT, and the tumor bed was delineated. In the case of persisting gross tumor after surgery and for the non-operated patients, a gross tumor volume (GTV) was defined by the tumor visualized on the planning CT and planning MRI. The clinical target volume (CTV) was defined as the GTV or the tumor bed with an additional margin (median 20 mm) dependent on histology and pathological features. In most cases, a boost dose to the high-risk area was delivered sequentially or as a simultaneous integrated boost (SIB). For the CTV boost, an additional margin (median 10 mm) was added to the GTV or the tumor bed, again dependent on the histology and pathological characteristics. The planning target volume (PTV) was defined as the CTV plus a 4–10-mm safety margin, depending on tumor location and patient immobilization. For PBSPT planning, a relative biological effectiveness (RBE) value of 1.1 was used.

### Follow-Up and Toxicity Assessment

Acute toxicity was recorded weekly during PT and assessed within the first 3 months after PBSPT. All subsequent institutional and external clinical notes were collected by our study and research office and reviewed during our weekly follow-up meeting to determine disease status and toxicity. All observed adverse events were graded according to the National Cancer Institute’s Common Terminology Criteria for Adverse Events, version 4.1 (CTCAE v4.1).

Local failure (LF) was either proven histologically or defined radiologically as residual tumor progression (an increase of ≥25% in size visible in MRI, CT, or PET-CT) or as the development of new nodular contrast enhancement and/or FDG uptake in the surgical bed compared to the baseline images. LFs occurring within the 95%, 50%–95%, and <50% isodose were classified as “in-field,” “marginal,” or “out-of-field,” respectively (17). Distant failure (DF) was defined as the development of new distant lesions in MRI, CT, or PET-CT follow-up or histologically proven by biopsy or resection.

### Statistical Analysis

Time to event data were calculated from the first day of PBSPT to the date of death or censored at the last follow-up using the Kaplan–Meier method. Death from any cause, LF, and DF were the defined events for the calculation of overall survival (OS), local control (LC), and distant control (DC), respectively. Group differences were analyzed with the log-rank test. Univariate Cox regression was used to investigate prognostic factors for LF, DF, and OS. Due to the cohort size and the limited number of events, a multivariate analysis was not deemed reasonable. Fisher’s exact test was used to compare toxicity rates in NF-1 and non-NF-1 patients. A p-value ≤0.05 was considered statistically significant. All statistical analyses were computed using SPSS version 26 (IBM, Armonk, NY, USA).

## Results

### Patient and Tumor Characteristics

Thirty-six patients with a median age of 32 years (range, 3–75) were included in this study, comprising 11 (31%) women and 25 (69%) men. Baseline characteristics are summarized in **Table 1**. Nine (25%) patients were younger than 18 years, and 15 (42%) patients were adolescents and young adults at the time of PBSPT. Histological confirmation was obtained for all patients. mPNST and PNST were diagnosed in 31 (86%) and 5 (14%) patients, respectively. Eight (22%) tumors were associated with underlying NF-1 disorder and 5 (14%) tumors (initial diagnosis: nasopharyngeal squamous cell carcinoma, n = 1; seminoma, n = 2; leukemia [total body irradiation], n = 1; and medulloblastoma [craniospinal irradiation], n = 1) were believed to be radiation induced because of the close proximity of the tumor with the previous radiation fields. About half of the tumors (n = 20, 56%) were located in the trunk, and two-thirds (n = 24, 67%) showed an initial local tumor extension of >5 cm. At the start date of PBSPT, all patients were assessed as non-metastatic. Histological workup of mPNSTs after complete resection (R0, n = 11, 31%) or partial resection (R1/R2 or biopsy, n = 15, 42%) showed FNCLCC (*Fédération Nationale des Centres de Lutte Contre Le Cancer*) Grades 1, 2, and 3 in 2 (6%), 14 (39%), and 10 (28%) cases, respectively. For 5 (14%) mPNST cases, there was insufficient information to assess an FNCLCC grade.

**Table 1:** Patient characteristics.

Characteristics	mPNST cohort (n = 31)	PNST cohort (n = 5)	All patients (n = 36)
**Age [years]**	31 (3–69)	39 (31–75)	32 (3–75)
**Pediatric (<18 years)**	9 (29)	0 (0)	9 (25)
**AYA (18–39 years)**	12 (39)	3 (60)	15 (42)
**Adults (>39 years)**	10 (32)	2 (40)	12 (33)
**Sex**			
**Female**	11 (35)	0 (0)	11 (31)
**Male**	20 (65)	5 (100)	25 (69)
**KPS or Lansky score**	90 (60–100)	90 (80–100)	90 (60–100)
**Neurofibromatosis type 1**	7 (23)	1 (20)	8 (22)
**Tumor radiation induced**	5 (16)	0 (0)	5 (14)
**Tumor localization**			
**Trunk**	19 (61)	1 (20)	20 (56)
**Extremities**	5 (16)	0 (0)	5 (14)
**Head and neck**	7 (23)	4 (80)	11 (30)
**Tumor size [cm]**			
**≤5**	9 (29)	3 (60)	12 (33)
**>5**	22 (71)	2 (40)	24 (67)
**Metastasis at time of RT**			
**Distant or nodal**	0 (0)	0 (0)	0 (0)
**FNCLCC Grade**			
**1**	2 (6)	–	2 (6)
**2**	14 (45)	–	14 (39)
**3**	10 (32)	–	10 (28)
**Unknown or NA**	5 (16)	5 (100)	10 (28)
**Resection status**			
**R0**	11 (35)	0 (0)	11 (31)
**R1**	4 (13)	1 (20)	5 (14)
**R2**	11 (35)	4 (80)	15 (41)
n **RX**	5 (16)	0 (0)	5 (14)

Values represent numbers (percent) or median (range) if not specified otherwise.

AYA, adolescents and young adults; KPS, Karnofsky performance score; RT, radiotherapy; R0, complete resection; R1, microscopic tumor residue; R2, macroscopic tumor residue; RX, no information on resection status; mPNST, malignant peripheral nerve sheath tumor; PNST, peripheral nerve sheath tumor; FNCLCC, Fédération Nationale des Centres de Lutte Contre Le Cancer.

### Treatment Characteristics

A summary of the treatment characteristics is detailed in **Table 2**. Eight (22%) patients received PBSPT for a recurrent tumor, whereas the majority of patients (n = 28, 78%) underwent PBSPT at diagnosis. Of the former group, 1 patient (3%) was treated previously with photon radiotherapy 42 months before PBSPT. PBSPT was delivered as adjuvant, neoadjuvant, and definitive treatments in 28 (78%), 5 (14%), and 3 (8%) cases, respectively. In total, 10 (28%) patients received chemotherapy as part of their treatment, as specified in **Table 2**. The median prescribed total dose was 64 Gy_RBE_ (range, 50–74) in 32 fractions (range, 17–39). Only 3 (8%) patients underwent combined irradiation with protons and photons: 2 patients (6%) received a boost with photons after completion of PT, and another patient (3%) started irradiation with photons and was boosted with protons. Most patients (n = 22, 61%) received a boost dose sequentially or as a SIB.

**Table 2:** Treatment characteristics.

Characteristics	mPNST cohort (n = 31)	PNST cohort (n = 5)	All patients (n = 36)
**PBSPT setting**			
**Primary treatment**	26 (84)	2 (40)	28 (78)
**Treatment of recurrence**	5 (16)	3 (60)	8 (22)
**PBSPT timing**			
**Adjuvant**	24 (77)	4 (80)	28 (78)
**Neoadjuvant**	5 (16)	0 (0)	5 (14)
**Definitive**	2 (6)	1 (20)	3 (9)
**Chemotherapy**			
**None**	21 (68)	5 (100)	26 (72)
**Prior to PBSPT**	3 (10)	0 (0)	3 (8)
**After PBSPT**	2 (6)	0 (0)	2 (6)
**Prior and after PBSPT**	1 (3)	0 (0)	1 (3)
**Prior to PBSPT and concomitant**	3 (10)	0 (0)	3 (8)
**Prior, concomitant and after PBSPT**	1 (3)	0 (0)	1 (3)
**Prescribed total dose [Gy_RBE_]**	66 (50–74)	51 (50–64)	64 (50–74)
**Total fractions**	33 (20–39)	27 (17–32)	32 (17–39)
**Single dose protons [Gy_RBE_]**	2 (1.8–3)	2 (1.8–3)	2 (1.8–3)
**Protons and photons combined**	3 (10)	0 (0)	3 (8)
**Photon fractions**	11 (10–25)	0	11 (10–25)
**Photon single dose (Gy)**	1.8	0	1.8
**Boost dose concept**			
**No boost**	9 (29)	5 (100)	14 (39)
**Sequential boost**	19 (61)	0 (0)	19 (53)
**SIB**	3 (10)	0 (0)	3 (8)
**PTV (cc)**	263 (28–2691)	44 (28–720)	232 (28–2691)
**V95_PTV (%)**	93 (46–100)	94 (81–99)	94 (46–100)

Values represent numbers (percent) or median (range) if not specified otherwise.

PBSPT, pencil beam scanning proton therapy; SIB, simultaneous integrated boost; PTV, planning target volume; V95_PTV, volume of the high-risk PTV that received 95% of the prescribed dose; mPNST, malignant peripheral nerve sheath tumor; PNST, peripheral nerve sheath tumor.

### Outcome

LF was observed in a total of 8 (22%; 1 PNST and 7 mPNST) patients, with 6 failures being classified as “in-field” and 2 others as “marginal” failures. Estimated 2-year LC rate was 73.5% (95% CI: 57.6%–89.4%). DF after PBSPT was observed in 14 (39%) patients, which resulted in an estimated overall 2-year DC rate of 61.2% (95% CI: 44.7%–77.7%). Sites of DF were the central nervous system (n = 5, 36%), lungs (n = 3, 21%), and soft tissue (n = 2, 14%). Four (29%) patients failed distantly at multiple sites. None of the PNST patients failed distantly. Analyzing only the mPNST cohort revealed an estimated 2-year DC rate of 56.7% (95% CI: 39.1%–74.3%) for this group. With a median follow-up time of 31 months (range, 4–194), 13 (36%) patients died from progressive mPNST disease. None of the patients with PNST had died. Overall, the estimated 2-year survival rates were 75.5% (95% CI: 60.6%–90.4%) and 72.8% (95% CI: 56.7%–88.9%) in the mPNST cohort.

**Figure 1:**
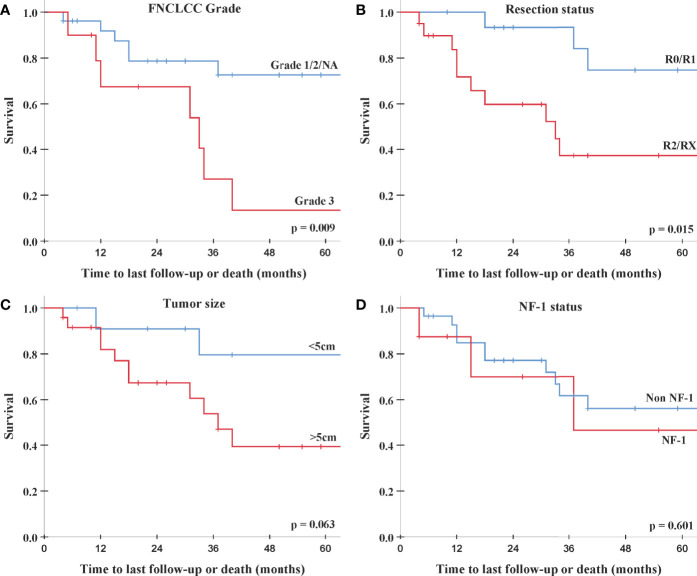
Log-rank analysis for overall survival. **(A)** FNCLCC Grade 1/2/NA (blue) vs. Grade 3 (red). **(B)** Resection status R0/R1 (blue) vs. R2/RX (red). **(C)** Tumor size <5 cm (blue) vs. >5 cm (red). **(D)** Non-NF-1 (blue) vs. NF-1 (red). FNCLCC, *Fédération Nationale des Centres de Lutte Contre Le Cancer*; NF-1, neurofibromatosis type 1.

On univariate analysis, no prognostic factor for LF was identifiable. However, univariate analysis showed a significant negative association between DF and higher FNCLCC grade (hazard ratio (HR) 3.79, 95% CI: 1.32–10.9, p = 0.013) and R2/RX resection status (HR 3.97, 95% CI: 1.1–14.3, p = 0.035, **Table 3**). These two factors demonstrate a similar impact on survival in univariate and log-rank analyses: the 2-year survival rates for patients with FNCLCC grade 3 tumors and R2/RX resection status were 67.5% (95% CI: 37.1%–97.8%) and 59.8% (95% CI: 36.7%–82.9%), respectively, while FNCLCC grade ≤2 tumors and R0/R1 resection status had 2-year survival rates of 78.7% (95% CI: 62%–95.4%) and 93.3% (95% CI: 80.8%–100%), respectively (**Figure 1** and **Table 3**).

**Table 3:** Univariate analysis.

Factors	Local failure (8 events)		Distant failure (14 events)		Survival (13 events)	
	HR (95% CI)	p-Value	HR (95% CI)	p-Value	HR (95% CI)	p-Value
**Gender** ** (female vs. male)**	1.51 (0.3–7.48)	0.616	0.83 (0.28–2.47)	0.732	1.01 (0.31–3.29)	0.987
**Age** ** (>32 vs. ≤32 years)**	1.07 (0.27–4.3)	0.921	1.03 (0.36–2.93)	0.960	1.38 (0.46–4.11)	0.567
**KPS/Lansky score** ** (≤80+NA vs. 90–100)**	1.44 (0.29–7.19)	0.658	2.97 (1.02–8.6)	0.045*	2.46 (0.8–7.55)	0.115
**FNCLCC Grade** ** (Grade 3 vs. rest)**	1.61 (0.38–6.74)	0.517	3.79 (1.32–10.9)	0.013*	3.99 (1.33–12.0)	0.014*
**Histology** ** (mPNST vs. PNST)**	0.82 (0.1–6.71)	0.853	25.3 (0.03–19874)	0.342	26.1 (0.03–22882)	0.345
**Tumor size** ** (>5 vs. ≤5 cm)**	4.22 (0.52–34.3)	0.179	3.85 (0.86–17.3)	0.079	3.78 (0.83–17.2)	0.085
**NF-1 status** ** (NF-1 vs. rest)**	1.43 (0.29–7.13)	0.662	1.13 (0.31–4.05)	0.855	1.41 (0.39–5.12)	0.605
**Tumor location** ** (extremities vs. trunk vs. head and neck)**	0.59 (0.22–1.59)	0.297	0.8 (0.42–1.52)	0.495	0.9 (0.47–1.72)	0.741
**Resection status** ** (R2+RX vs. R0+R1)**	2.09 (0.5–8.85)	0.315	3.97 (1.1–14.3)	0.035*	4.37 (1.19–16.0)	0.026*
**Chemotherapy** ** (CT vs. no CT)**	3.26 (0.81–13.1)	0.096	2.04 (0.71–5.9)	0.189	2.54 (0.85–7.6)	0.097
**PTV size high risk** ** (>231.9 vs. ≤231.9 cc)**	2.53 (0.49–13.1)	0.267	2.28 (0.76–6.82)	0.140	1.65 (0.52–5.21)	0.393

HR, hazard ratio; KPS, Karnofsky performance status; FNCLCC, Fédération Nationale des Centres de Lutte Contre Le Cancer; (m)PNST, (malignant) peripheral nerve sheath tumor; NF-1, neurofibromatosis type 1; CT, chemotherapy; PTV, planning target volume.

^*^Statistically significant.

Additionally, on univariate analysis lower performance score (KPS/Lansky ≤ 80) was significantly associated with increased DF, and patients with larger tumors (>5 cm) showed a trend toward an increased risk for DF and worse survival. NF-1 patients had a similar failure and survival rates as non-NF-1 patients (**Table 3** and **Figure 1**).

### Toxicity

Acute grade 1 dermatitis was the most common acute toxicity (n = 20, 56%). Acute grade 2 dermatitis, conjunctivitis, and mucositis were seen in 8 (22%), 1 (3%), and 1 (3%) patients, respectively. In 4 (11%) patients, acute grade 3 dermatitis was observed, while 1 (3%) patient presented with acute grade 3 mucositis, and 1 (3%) patient developed grade 3 pain in the irradiated extremity.

All observed late toxicities are detailed in **Table 4**. Overall, late toxicity was noted in 16 (44%) patients, mostly as grade 1 hyperpigmentation (n = 6, 17%). Out of these 16 patients, 5 (14%) presented with multiple late toxicities. Late grade 2 toxicity was observed in 4 (11%) patients as otitis media (n = 1, 3%), nasal crusting (n = 1, 3%), musculoskeletal deformity (n = 1, 3%), and hyperpigmentation (n = 1, 3%). Of these 4 patients, later on, one (3%) patient developed two late grade 3 toxicities (cataract and osteoradionecrosis) 34 and 194 months after PBSPT. Of note, this non-NF-1 patient was treated 11 years before PBSPT for a nasopharyngeal tumor with 70 Gy. Two-year late grade >3 toxicity rate was 0%. There was no observed grade 4 or 5 toxicity or radiation-induced secondary cancer.

**Table 4:** Summary of late toxicities according to CTCAE v4.1 in alphabetical order.

Toxicity	Grade 1 n (%)	Grade 2 n (%)	Grade 3 n (%)	Grade 4 n (%)	Grade 5 n (%)
**Alopecia**	2 (6)				
**Cataract**	1 (3)		1 (3)		
**Crusting nasal**	1 (3)	1 (3)			
**Fibrosis**	2 (6)				
**Hoarseness**	1 (3)				
**Hyperpigmentation**	6 (17)	1 (3)			
**Musculoskeletal deformity**		1 (3)			
**Otitis media**		1 (3)			
**Radiation necrosis**	2 (6)		1 (3)		
**Trismus**	1 (3)				
**Xerostomia**	1 (3)				
**Total**	17 (47)	4 (11)	2 (6)	0 (0)	0 (0)
** Non-NF-1 (n = 28)**	14 (50)	3 (11)	2 (7)	0 (0)	0 (0)
** NF-1 (n = 8)**	3 (38)	1 (13)	0 (0)	0 (0)	0 (0)

Values represent numbers (percent). Multiple toxicities were observed in 5 patients.

NF-1, neurofibromatosis type 1; CTCAE, Common Terminology Criteria for Adverse Events.

The rate of any grade ≥3 acute toxicity and any late toxicity in NF-1 patients was 25% and 50% and for non-NF-1 patients 11% and 43%, respectively. These differences in toxicity rates in NF-1 and non-NF-1 patients did not translate into statistical significance using Fisher’s exact test (p = 0.305 for any grade ≥3 acute toxicity and p = 0.514 for any late toxicity).

## Discussion

In this study, we analyzed the clinical outcome of non-metastatic patients with PNSTs and mPNSTs treated with PBSPT. To the best of our knowledge, this is the first report of the oncological outcome and toxicity rates of mPNST/PNST patients treated with protons using a PBS delivery paradigm. Of note, two retrospective studies and two case reports analyzing mPNSTs treated with carbon ion (C12) particles were published (18–20). Jensen et al., 2015 (18) reported on the outcome and toxicity of 11 patients with unresected or incompletely resected mPNSTs treated with C12 irradiation. Patients with combined photon irradiation received 50 Gy photon intensity-modulated radiation therapy (IMRT) in 25 fractions and a 24 Gy_RBE_ boost with C12 irradiation in 8 fractions, while patients treated solely with C12 irradiation received 60–66 Gy_RBE_ in 20–22 fractions. With a median follow-up of 17 months, the authors reported a 2-year LC, progression-free survival, and survival rates of 65%, 56%, and 75%, respectively. Similar results were observed by Vitolo et al., 2019 (19) in their retrospective series of 13 patients with unresected mPNSTs treated with C12 irradiation (median dose of 73.6 Gy_RBE_ in 16 fractions) and a median follow-up of 24.6 months: reported 2-year LC and survival rates were 63% and 60%, respectively. Compatible with the C12 literature, we report in our cohort consisting mainly of patients with mPNSTs 2-year LC, DC, and OS rates of 73.5%, 61.2%, and 75.5%, respectively (**Figure 1**). The superior LC rate in our study is possibly due to the fact that in both C12 studies, mainly patients with gross residual tumors were included. While prospective studies examining the impact of irradiation for PNST and mPNSTs are still missing, some larger retrospective series are available. The outcome of the selected studies and the two aforementioned C12 studies is summarized in **Table 5**. One of the biggest series was published by Bishop et al., 2018 (22). These authors analyzed 71 mPNSTs treated with external beam radiation therapy (EBRT) either preoperatively with a median dose of 50 Gy or postoperatively with a median dose of 64 Gy. They reported excellent 5-year LC and survival rates of 84% and 66%, respectively (**Table 5**). However, patients with recurrent and radiation-induced tumors, which are known to have the worse outcome (1, 25–28), were excluded from their analysis. Furthermore, they found positive/uncertain surgical margin status to be adversely associated with local recurrence at 5 years (28% vs. 5% for negative margins, p = 0.02).

**Table 5:** Summary of studies detailing the outcome after irradiation of mPNSTs.

Authors	n	FU [months]	Histology and NF-1 status	Irradiation	Dose [Gy/Gy_RBE_, median]	LC	OS	G° ≥ 2 Tox and 2nd TU
**Wong et al., 1998** (21)	73*	53	mPNST NF-1: 24%*	EBRT (59%), Brachy (19%), IOERT (22%)	EBRT: 50.7 Brachy: 15 IOERT: 12.5 Combined: 60	5 years: 65%	5 years: 58%	–
**Bishop et al., 2018** (22)	71	118	mPNST NF-1: 37%	Pre-OP (32%), post-OP (68%) EBRT	Pre-OP: 50 Post-OP: 64	5 years: 84%	5 years: 66%	3%
**Kahn et al., 2014** (23)	20^§^	–	mPNST NF-1: 50%	EBRT (80%), Brachy (10%), Brachy+EBRT (10%)	EBRT: 58.5–59.4	5 years: 53%	5 years: 44%^§^	15% 2nd TU
**Sloan et al., 2018** (24)	15	29	mPNST NF-1: 100%	EBRT post-OP (46%), Pre-OP (47%), definitive (7%)	50 (all patients)	5 years: 91%	5 years: 53%	13% 2nd TU
**Vitolo et al., 2019** (19)	13	24	mPNST NF-1: -	Post-OP C12 (100%)	73.6	2 years: 63%	2 years: 60%	15%
**Jensen et al., 2015** (18)	11	17	mPNST	IMRT+C12 (27%), C12 alone (73%)	IMRT+C12: 74 C12 alone: 60	2 years: 65%	2 years: 75%	18%
**Present study**	36	31	mPNST (86%) PNST (14%) NF-1: 22%	PBSPT post-OP (78%), pre-OP (14%), definitive (9%)	64 (all patients)	2 years: 74% 5 years: 74%	2 years: 76% 5 years: 54%	11%

n, number of patients; NF-1, neurofibromatosis type 1; LC, local control; OS, overall survival; Tox, late toxicity; 2nd TU, secondary tumors; (m)PNST, (malignant) peripheral nerve sheath tumor; EBRT, external beam radiation therapy; Brachy, brachytherapy; IOERT, intra-operative electron radiotherapy; C12, carbon ion irradiation; IMRT, intensity-modulated radiotherapy; PBSPT, pencil beam scanning proton therapy.

^*^73/134 patients received RT, and 24% of all patients had NF-1.

^§^20/33 patients received irradiation, and the 5-year OS rate of all patients was 44%.

In our cohort, univariate analysis revealed no factor to be significantly associated with LF. Patients with FNCLCC Grade 3 mPNSTs were however significantly more likely to develop distant metastases (HR 3.79, 95% CI: 1.32–10.9, p = 0.013; **Table 3**) and to die (HR 3.99, 95% CI: 1.33–12.0, p = 0.014; **Table 3**). For patients with macroscopic or uncertain resection status (R2/RX), a similar circa 4-fold risk increase to develop DFs (HR 3.97, 95% CI: 1.1–14.3, p = 0.035; **Table 3**) and decrease (HR 4.37, 95% CI: 1.19–16, p = 0.026; **Table 3**) was observed. Furthermore, on univariate analysis, a performance status score of ≤80 was negatively associated with DF (p = 0.045), and a trend toward increased DF and worse survival was seen for tumors larger than 5 cm (see **Table 3**). Tumor location seemed to have no impact on outcome in our cohort. These findings are largely in line with the existing literature where higher tumor grade (21, 23, 27, 29–33), incomplete resection (11–13, 23, 26, 27, 29, 31, 34), larger tumor size (11, 12, 21, 25, 27, 30–35), truncal tumors (12, 23, 25, 26, 30) and decreased performance status (36) were shown to be associated with worse prognosis.

There is some conflicting information in the literature concerning the impact of NF-1 disorder in mPNST patients. The majority of studies report decreased survival for mPNSTs occurring in NF-1-patients (11, 13, 26, 27, 29, 30), while several other studies report similar outcomes for NF-1 associated mPNSTs (12, 25, 31, 34). More recent studies have shown that genetically NF-1 and non-NF-1 mPNSTs seem to be indistinguishable (37, 38), and so far, no determining molecular differences have been identified (31). Most authors, therefore, attribute the observed poorer outcome of NF-1-associated mPNSTs to several accompanying factors known to be associated with worse prognosis, namely, 1) mPNSTs in NF-1 patients tend to be larger in size at the time of diagnosis (13, 26, 30), 2) the tumors are often in a non-extremity location and are therefore less amenable to surgery (11, 39), 3) NF-1 patients are more likely to develop other malignancies that might impair survival (13), and finally, 4) NF-1-associated tumors seem to respond less to chemotherapy (13). In 2013, Kolberg et al. (31) published survival meta-analyses comprising >1,800 mPNSTs comparing the survival of NF-1 and non-NF-1 patients. Indeed, they found that non-NF-1 patients had significantly increased OS (HR 1.38, 95% CI: 1.1–1.72, p = 0.004) and disease-specific survival rates (HR 1.4, 95% CI: 1.13–1.75, p = 0.002). Interestingly, this observed difference in survival is diminishing when only analyzing the patients treated after the year 2000. While disease-specific survival rates remain borderline significantly increased for non-NF-1 patients (HR 1.32, 95% CI: 1.0–1.74, p = 0.05), OS for NF-1 patients treated in the last 2 decades did not differ significantly compared to non-NF-1 patients (HR 1.19, 95% CI: 0.85–1.66, p = 0.3). A similar survival improvement for more recently treated NF-1-associated mPNSTs has been observed by Ingham et al., 2011 (40). Kolberg et al. (2013) considered that increased awareness among NF-1 patients and improved monitoring routines in the last years have led to earlier detection of mPNSTs and, thus, increased survival in NF-1 patients. One might also speculate that in the past, NF-1-associated mPNSTs were treated differently and that nowadays, due to improved and safer treatment modalities, NF-1 patients benefit from the same anticancer treatment as non-NF-1 patients.

In our cohort, NF-1 disorder was not associated with worse oncological outcomes (**Table 3** and **Figure 1**). On the one hand, only 8 (22%) patients had proven NF-1 disorder, which rendered finding a statistical difference for this sub-cohort difficult. On the other hand, 35 (97%) of our patients were treated after the year 2000 and 28 (78%) after the year 2010.

Cells from patients harboring a mutated NF-1 gene are considered to be more radiosensitive. No significant rate of relevant acute or late toxicity was observed in our NF-1 patients. In general, we report very low toxicity rates for our cohort after PBSPT. Late grade ≥2 toxicity rate was observed in 4 (11%) patients. There was no observed late grade 4 or 5 toxicity. These rates are in line with the reported toxicity in the literature, particularly when comparing PBSPT with C12 irradiation (**Table 5**). Jensen et al. (2015) (18) reported late grade 3 toxicity in 2 (18%) patients, and Vitolo et al. (2019) (19) observed late grade ≥2 toxicity in 2 (15%) patients. No secondary tumor after PBSPT was observed in our cohort, although the follow-up period of our series is short. Kahn et al. (2014) (23) and Sloan et al. (2018) (24) reported in their series secondary tumor rates of 15% and 13%, respectively. However, their cohorts consisted of 50% and 100% NF-1 patients, which highlights the concern of secondary tumors after irradiation for this selected patient group.

Undoubtedly, our study has certain limitations, mainly being retrospective in nature with its inherent biases. A larger sample size and a longer follow-up period would have further increased the clinical validity of this study. The small sample size of 36 patients limited the statistical power to detect associations between outcome and some of the clinical factors examined. Our inability to perform a multivariate analysis also prohibits definitive conclusions regarding the outcome and prognostic factors. Additionally, this study consists of a rather heterogeneous cohort, including patients of any age, any resection status, and any histologic grade as well as five PNST cases. However, PNSTs are known to be a precursor of mPNSTs and are sometimes difficult to distinguish from mPNSTs (2, 10, 41, 42). As such, PNSTs usually require the same multimodal treatment including surgery and irradiation due to their destructive growth.

## Conclusion

In our cohort, consisting mainly of patients with mPNSTs, we report 2-year LC and OS rates of 73.5% and 75.5%, respectively. The majority (n = 14, 39%) of patients failed distantly, and FNCLCC grade and resection type were significantly associated with DF. Only 1 (3%) patient presented with high-grade toxicity. No difference in toxicity rates was observed between NF-1 and non-NF-1 patients. Additional PT series are needed to legitimate the administration of protons in these young challenging patients.

## Data Availability Statement

The raw data supporting the conclusions of this article will be made available by the authors, without undue reservation.

## Ethics Statement

The studies involving human participants were reviewed and approved by Ethikkommission Nordwest- und Zentralschweiz (2021-00369). Written informed consent to participate in this study was provided by the participants’ legal guardian/next of kin.

## Author Contributions

NB established the concept and design of the study; acquired, analyzed, and interpreted the data; and drafted the article. DL supported the statistical analysis and critically reviewed the submitted version of the manuscript. AP critically reviewed the submitted version of the manuscript. BB critically reviewed the submitted version of the manuscript. DW established the concept and design of the study, supervised the study, interpreted the data, and critically reviewed the submitted version of the manuscript. All authors listed have made a substantial, direct, and intellectual contribution to the work and approved it for publication.

## Funding

Open access funding provided by PSI - Paul Scherrer Institute.

## Conflict of Interest

The authors declare that the research was conducted in the absence of any commercial or financial relationships that could be construed as a potential conflict of interest.

## Publisher’s Note

All claims expressed in this article are solely those of the authors and do not necessarily represent those of their affiliated organizations, or those of the publisher, the editors and the reviewers. Any product that may be evaluated in this article, or claim that may be made by its manufacturer, is not guaranteed or endorsed by the publisher.
